# Secondary Dentin Formation Mechanism: The Effect of Attrition

**DOI:** 10.3390/ijerph18199961

**Published:** 2021-09-22

**Authors:** Itay Nudel, Ariel Pokhojaev, Yoli Bitterman, Nir Shpack, Luca Fiorenza, Stefano Benazzi, Rachel Sarig

**Affiliations:** 1 Department of Oral Biology, The Maurice and Gabriela Goldschleger School of Dental Medicine, Sackler Faculty of Medicine, Tel Aviv University, Tel Aviv 6997801, Israel; inudel1@gmail.com (I.N.); pokhojaev@gmail.com (A.P.); dr.bitterman@gmail.com (Y.B.); 2 Department of Orthodontics, The Maurice and Gabriela Goldschleger School of Dental Medicine, Sackler Faculty of Medicine, Tel Aviv University, Tel Aviv 6997801, Israel; nshpack@post.tau.ac.il; 3 Department of Anatomy and Developmental Biology, Monash Biomedicine Discovery Institute, Monash University, Clayton, VIC 3800, Australia; luca.fiorenza@monash.edu; 4 Department of Cultural Heritage, University of Bologna, 48121 Ravenna, Italy; stefano.benazzi@unibo.it; 5 The Dan David Center for Human Evolution and Biohistory Research, Shmunis Family Anthropology Institute, Sackler Faculty of Medicine, Tel-Aviv University, Tel Aviv 6997801, Israel

**Keywords:** dentin, dental anatomy, finite element analysis (FEA), micro-computed tomography, mastication

## Abstract

Human dentin consists of a primary layer produced during tooth formation in early childhood and a second layer which first forms upon tooth eruption and continues throughout life, termed secondary dentin (SD). The effect of attrition on SD formation was considered to be confined to the area subjacent to attrition facets. However, due to a lack of three-dimensional methodologies to demonstrate the structure of the SD, this association could not be determined. Therefore, in the current study, we aimed to explore the thickening pattern of the SD in relation to the amount of occlusal and interproximal attrition. A total of 30 premolars (50–60 years of age) with varying attrition rates were evaluated using micro-computerized tomography. The results revealed thickening of the SD below the cementoenamel junction (CEJ), mostly in the mesial and distal aspects of the root (*p* < 0.05). The pattern of thickening under the tooth cervix, rather than in proximity to attrition facets, was consistent regardless of the attrition level. The amount of SD thickening mildly correlated with occlusal attrition (*r* = 0.577, *p* < 0.05) and not with interproximal attrition. The thickening of the SD below the CEJ coincided with previous finite element models, suggesting that this area is mostly subjected to stress due to occlusal loadings. Therefore, we suggest that the SD formation might serve as a compensatory mechanism aimed to strengthen tooth structure against deflection caused by mechanical loading. Our study suggests that occlusal forces may play a significant role in SD formation.

## 1. Introduction

The inner dentin layer surrounding the pulp chamber shows continued, age-correlated obliteration of the pulp chamber [1,2]. Pulp reduction by secondary dentin (SD) formation is initiated from apex closure throughout life, by the same odontoblast cells that form the primary dentin (PD) [3,4]. The histological differentiation between SD and PD is based on a shift in tubule direction between the two layers [4,5] and small differences in ground substance organization [6]. Secondary dentin formation first occurs at the pulp chamber’s most coronal aspects, in a nonhomogeneous manner that may change with the examined tooth type [3,7]. Moreover, caries and restorative procedures cause odontoblast or odontoblast-like cells to create a third form of unorganized dentin, termed tertiary dentin (also referred to as reparative dentin) [4]. The minute histological difference may result in biased unification of SD and tertiary dentin. Thus, the formation of SD itself is sometimes considered to be affected by erosion, caries, or changes in osmotic pressure throughout the pulp chamber [8].

Attrition, created by tooth-to-tooth wear over a lifetime of mastication, was suggested to affect the formation of SD. Attrition can affect occlusal surfaces [9,10] and inter-proximal surfaces created by the contact of adjacent teeth during mastication and function [11,12].

The reactionary dentine was observed predominately in aged, attired teeth, which might affect the low hardness and modulus of elasticity of this area [13]. It has been suggested that the atubular structures provide a defense mechanism against physiological attrition that reduces the dentine permeability [14]. Moreover, it has been argued that this mechanism is an important factor in protecting the pulp from noxious irritation [15].

However, the exact effect of attrition remains unclear. Philippas et al. showed very little correlation between attrition and SD formation, whereas Burke et al. and Tronstad et al. obtained contradicting results [3,5,16]. Of note, the observations made for SD formation have used histological slices, which allow for separation between dentin layers, although are confined to two dimensions for each slide.

The need to elucidate the effect of attrition on SD formation is evident when considering the pulp obliteration process. This might impair the ability to perform root canal irrigation and disinfection in elderly patients [5,7]. Moreover, the effect of attrition should be noted when considering the use of SD for age estimation. Several methods, ranging from periapical X-rays and orthopanograms to three-dimensional CBCTs, were adopted for forensic age estimation based on pulp volume constriction [1,2,7,17,18].

The aim of this study was to analyze the association between attrition and SD formation by analyzing teeth with varying degrees of attrition from a cohort of same-aged individuals. Applying a novel technique to segment the SD using micro-tomography [19], we examined the SD architecture in three dimensions, allowing us to discern the effects of attrition while omitting confounders such as age and sex. By doing so, we suggest a new hypothesis for the formation mechanism of SD, providing basic understanding for the tooth-aging process and dental physiology.

## 2. Materials and Methods

### 2.1. Materials

The dental material used in the study was retrieved from the Hamann-Todd Osteologic Collection (Laboratory of Physical Anthropology at the Cleveland Museum of Natural History, Cleveland, OH, USA. Hereinafter, HTH). The sample was composed of 30 premolars from Caucasian males aged between 50 and 60 years (average 55 ± 3 years) of European–American descent. All subjects were born between 1825 and 1910, and were mostly from low socio-economic status. For each individual, a mandibular premolar was scanned and selected based on the ability of removal without harming the specimen. The inclusion criteria were: intact teeth without caries, restorations, root fillings, or severe fragments. Data regarding their estimated age at death (recorded in whole years), sex, and ethnicity were obtained from the HTH collection records.

### 2.2. Methods

#### 2.2.1. Micro CT Scan

Teeth were scanned using Bruker Skyscan 1172 (Bruker MicroCT, Kontich, Belgium) with the following parameters: 17.14 μm voxel size, 80 kV, 100 μA, 0.5 aluminum, and 0.04 mm copper filters applied over 360 degrees of rotation with acquisition every 0.3 degrees. Batched teeth scans were segmented to single teeth files in 3D TIFF format using Amira 6.2 (Thermo Fisher Scientific, Waltham, MA, USA).

#### 2.2.2. Micro CT Analysis

**Figure 1:**
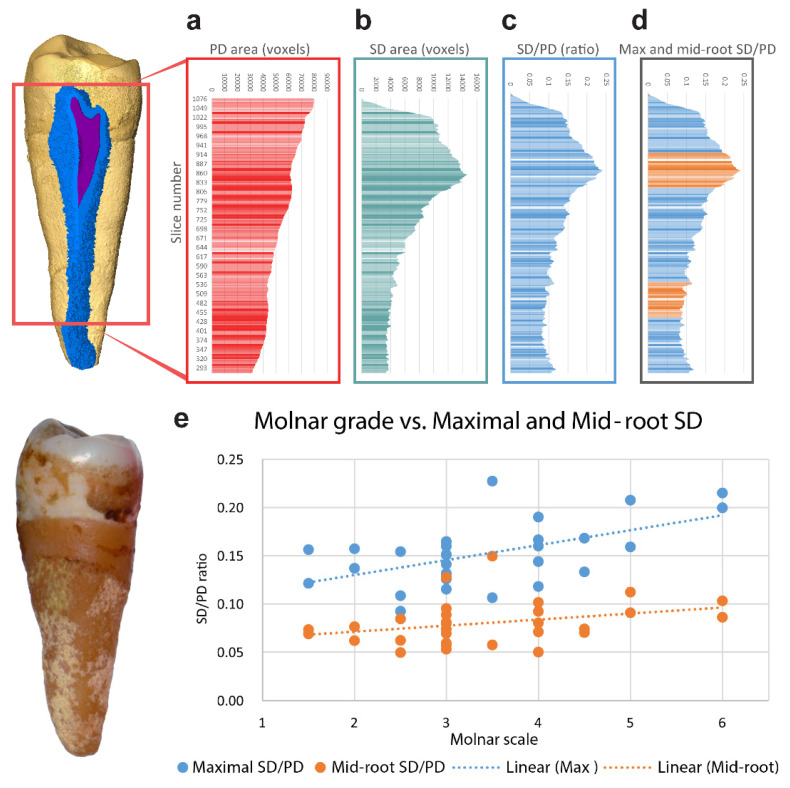
SD and PD evaluation of horizontal slices. Representative image of 30 teeth examined, isosurface of a tooth with a three-dimensional representation of the SD layer (blue) on top of the pulp (purple) and original tooth below. (**a**) PD area in voxels (red) for each slice from the first slice with SD to the bottom third of the tooth. (**b**) SD area (green) for each voxel slice. (**c**) SD/PD ratio (blue) for each slice. (**d**) Max and mid-root SD/PD ratios. There are 100 maximal (upper) and 100 mid-root slices (lower) demarcated in orange. (**e**) SD/PD values of maximal (blue) and mid-root (orange) measurements, partitioned by attrition levels (*r* = 0.577 * and *r* = 0.331 accordingly, Spearman’s correlation) * *p* < 0.05, *n* = 30.

Secondary dentin segmentation was conducted using Amira 6.2 after tooth orientation based on the CEJ contour, derived from landmarks digitally placed on the scanned tooth, according to Benazzi et al. [20]. Differentiation threshold values for SD were examined by histogram after non-local means filtering, applied to emphasize the SD border. Segmentation was based on a watershed algorithm, as elaborated in Nudel et al. [19]. Briefly, using threshold partitioning, enamel, dentin, and pulp were segmented. Watershed was applied after digital removal of outer voxels (erosion) and, consequently, filling the created voids. An SD layer adjacent to the pulp was added after applying a second erosion on dentin, and dilation of the pulp layer voxels, while locking the validated pulp from manipulation. Manual corrections were sparsely used due to some coronal cracks creating radiolucent areas adjacent to the SD border. One millimeter from the tooth apex was omitted due to the effect of erosion dilation procedures on the constricted apical part of the tooth, which skewed the shape of the terminal ends (Figure 1d).

#### 2.2.3. SD Analysis

Dentin layers were examined using horizontal slices at each voxel height (17 microns) for SD and PD using an ImageJ script (National Institutes of Health, USA) on the segmented teeth. The number of voxels of SD and PD for each slice was exported for analysis. The analysis focused on two measurements: maximal SD/PD and mid-root SD/PD ratios. Maximal SD/PD represents the average of ratios from 100 slices above and below the slice with maximal thickening (identified using MAX function, Excel 2016). Mid-root SD/PD represents a similar average from 100 slices at the mid-root, defined based on a count from the first slice with SD to the bottom apical millimeter of the tooth. Maximal SD/PD and mid-root SD/PD values were calculated for each tooth.

The segmented SD layer was also visualized three-dimensionally by generating a thickness map of the entire segmented layer using Amira 6.2. All thickness maps were displayed using a “physics” color scheme ranging from 0 to 0.75 mm. A bounding box of 1 mm was displayed extending 1mm below the CEJ to facilitate orientation in the vertical dimension. The vertical location of maximal SD thickening was evaluated by placing a line probe on the mesial area. A range of slices with maximal thickening was transferred for statistical analysis together with the CEJ slice kept as a reference. Measurements of SD thickening for mesial, distal, lingual and buccal were conducted on an orthogonal slice 1 mm below the CEJ, with the SD border overlaid (“color wash” module).

Age-related SD accumulation was compared using a 1 mm slice below the CEJ and evaluating the SD/PD ratio, as elaborated in Nudel et al. [19].

#### 2.2.4. Attrition

The attrition level was ranked based on Molnar’s grade of occlusal attrition with the addition of half grades [9]. Attrition levels 1.5 to 3 designated increasing enamel wear without dentin patches, whereas 3.5 to 6 ranged from small dentin patches to the complete loss of cusps leaving only a surrounding enamel ring. Ranks were assigned by two researchers independently and later compared (RS, IN). Of note, attrition and abrasion were considered together due to their phenotypic similarity, herein referred to as attrition [21]. Interproximal wear facets were measured using an area probe module, encompassing the facets while exporting the two-dimensional area for statistical analysis. Measurements were conducted by two researchers separately (RS, IN) and compared.

### 2.3. Statistical Analysis

All statistical analysis was conducted using the SPSS 22 software (IBM, Armonk, NY, USA).

Attrition levels and interproximal facets were ranked by two separate inspectors (RS, IN), and inter-observer differences were evaluated using the intraclass correlation coefficient (ICC). ICC values were assessed according to Cicchetti 1994 [22].

The initial sample size calculation was based on previous research of low-attrition subjects [19]. Given a standard deviation within a ten-year age group of 0.03 for SD/PD, and a 0.055 clinically significant difference (indicating a relevant change for forensic age estimates [23]), to achieve an 80% power with an α of 0.05, the required sample of high-attrition subjects was 10.

Age-related changes were evaluated using an independent samples Student’s *t*-test.

The relation between occlusal attrition and SD formation was examined using Student’s *t*-test between two groups of low and high attrition (1.5–3 and 3.5–6 Molnar attrition level, independent samples). Correlation between attrition and SD formation was examined using Spearman’s correlation. The vertical distribution of maximal SD thickening was examined using both a chi-squared test and Spearman’s correlation.

Evaluation of SD thickening for mesial, distal, buccal and lingual directions was based on paired samples Student’s *t*-test. Results were considered significant at *p* < 0.05. The correlation between mesial and distal wear facets and corresponding SD thickening was evaluated using the Pearson correlation.

## 3. Results

Thirty mandibular premolars from males of a similar age group were ranked in order to examine the relationship between occlusal attrition and SD formation. The occlusal attrition ranged from mild to severe (1.5 to 6), with an average of 3.4 ± 1.1 (Table 1). Attrition levels were ranked by two separate inspectors with a high inter-observer agreement (ICC = 0.829, single measures, absolute agreement, *p* < 0.001).

Measuring horizontal slices at each voxel height after segmenting SD and PD revealed that the PD continuously decreased towards the apex (Figure 1a), whereas the SD showed a different pattern (Figure 1b). The SD at the mid-root of the tooth remained mostly continuous with a small reduction toward the apex, whereas the area below the crown cementoenamel junction (CEJ) showed thickening of the SD. The SD/PD ratio showed a similar pattern to the SD (Figure 1c) and was chosen as a parameter that accounted for premolar size discrepancy.

The maximal SD/PD thickening was significantly different between groups of low and high attrition (1.5–3 and 3.5–6 grades concomitantly, independent samples Student’s *t*-test, *p* = 0.036). Measuring mid-root SD/PD showed only a slightly higher ratio in the high-attrition group, which was not significantly different (Figure 1d). Maximal thickening showed a mild significant correlation with attrition (*r* = 0.577, *p* = 0.003, Spearman’s correlation), whereas the mid-root area did not show a significant correlation (Figure 1e). Post hoc analysis of the results indicated a power of 0.76.

**Figure 2:**
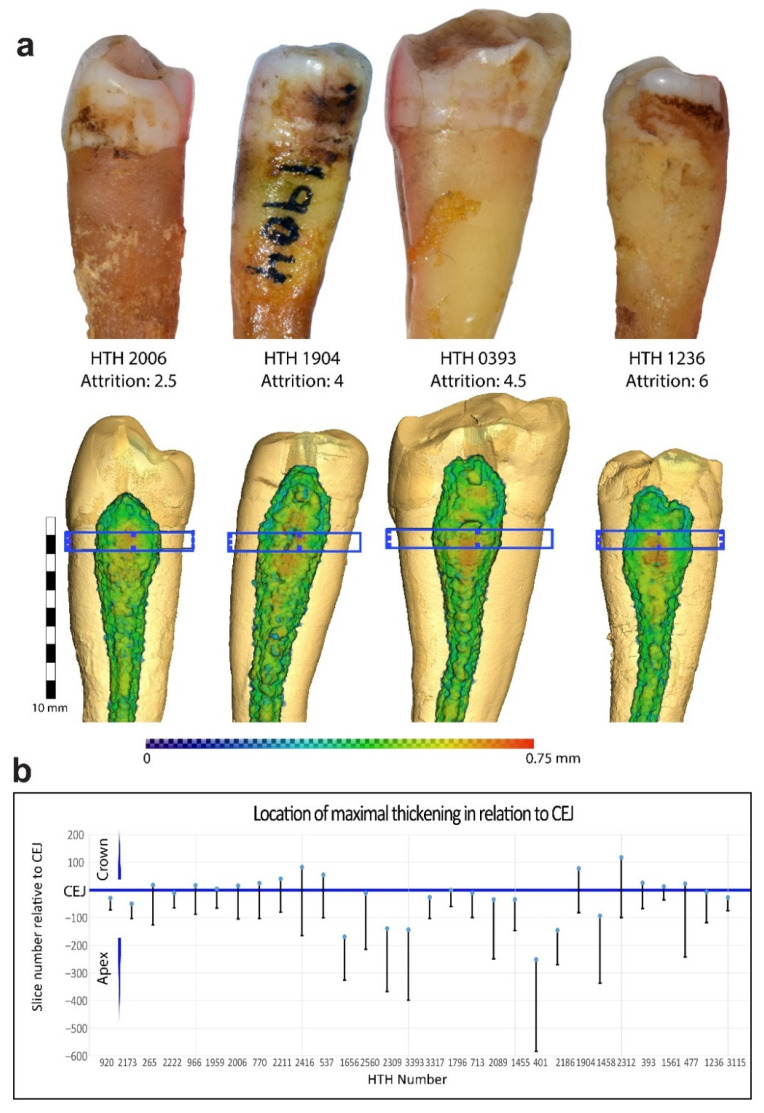
Three-dimensional thickness map. (**a**) Images of four teeth (on top) and isosurface of the teeth with a three-dimensional thickness map overlaid (below). A blue box indicates a 1 mm slice below the CEJ. The bottom legend indicates SD thickness ranging from 0 to 0.75 mm. (**b**) Vertical distribution of maximal SD thickening. The blue line indicates CEJ level, and the bars indicate maximal thickening for each tooth. Slice numbers are relative to CEJ. *n* = 30.

**Figure 3:**
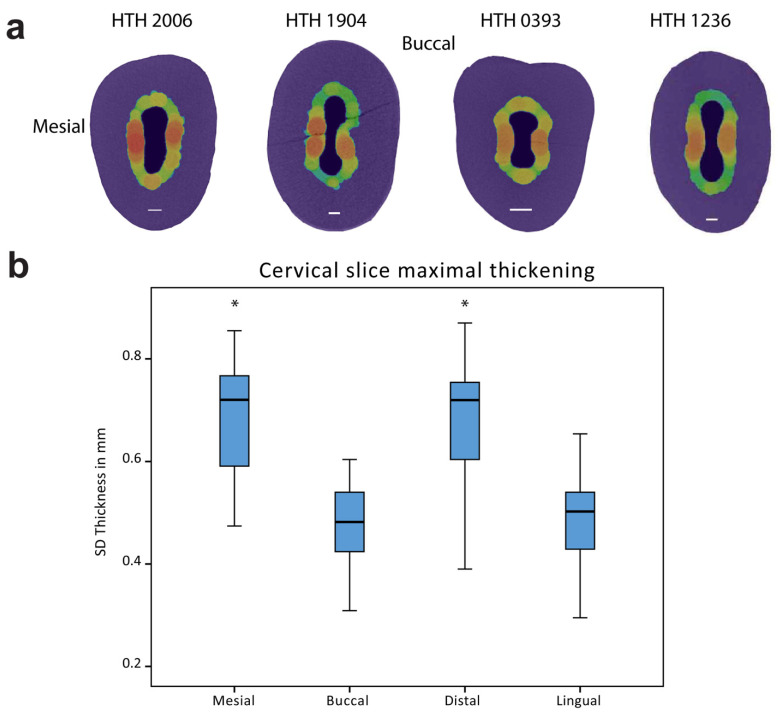
Mesio-distal thickening of SD. (**a**) Horizontal slices at −1 mm below the CEJ from the four representative teeth. Buccal and mesial directions are on the top and on the right, respectively. SD thickness values are based on the thickness legend. (**b**) Box plot of the SD thickness 1 mm below the CEJ at mesial, buccal, distal, and lingual directions. *n* =30. * Indicating significant difference at *p* < 0.05. Significance was found between the mesial and distal to buccal and lingual.

Three-dimensional evaluation of SD was based on a thickness map of the entire segmented SD layer. The generated map showed prominent thickening appearing below the tooth CEJ (Figure 2a,b). No correlation was found between the vertical maximal SD location and the amount of attrition (the chi-squared test and Spearman’s correlation were both non-significant). Evaluating the cervical thickening showed an increase in both mesial and distal surfaces, which was significantly greater when compared to the buccal or lingual surfaces (paired samples Student’s *t*-test, *p* < 0.001, Figure 3a,b). The amount of SD thickening in mesial and distal sides was not correlated to the size of the corresponding attrition facets (*r* = 0.072 and *r* = −0.16 accordingly, both non-significant, Pearson correlation).

## 4. Discussion

The effect of attrition on SD formation was analyzed for the first time using a three-dimensional display of the SD architecture. By examining a homogeneous group of ages, we could eliminate the age factor, which is a crucial confounding variable that affects SD formation. Using a same-sex group, another possible confounder was negated, even though previous work suggests that SD formation is not affected by sex, and that gender is not a considerable confounder [3,7,8,19]. Segmented SD was evaluated using two-dimensional measurements at each horizontal slice and three-dimensional thickness maps; both showed a similar pattern of SD thickening below the CEJ. This pattern was evident in the vast majority of the teeth, regardless of the attrition level. However, the amount of thickening (SD/PD ratio) correlated with higher attrition levels.

Reviewing dentin accumulation in previous attrition studies indicated a location subjacent to wear facets. Tronstad et al. reported on severe attrition in deciduous incisors creating unorganized reparative “irritation dentin” [16]. A similar accumulation of irregular dentin was noted by Philippas et al. in their work on maxillary incisors [3]. These phenomena were not evident in our sample subjacent to occlusal wear facets. This could probably indicate that the formation of SD results from force loadings rather than from the attrition effect, since the pattern of stress distribution in incisors is directed differently. Supporting this notion, Tanaka et al. examined stress distribution and showed that tensile stress in maxillary incisors is directed from the CEJ upwards toward the cusp tips, whereas mandibular premolars display tensile stress more horizontally below the CEJ [24]. Similar to premolars, finite element analysis (FEA) conducted for molars indicated a pattern of stress distribution below the CEJ [25], emphasizing the possible connection between occlusal forces and SD formation at this area.

Increased thickening has been seen in the mesial and distal faces of the pulp chamber, which are adjacent to the interproximal contacts with adjacent teeth. A lack of correlation with the size of attrition facets was evident, further indicating that SD thickening is less affected by tooth wear. FEA examination indicates the transfer of load to the mesial and distal surfaces in various loading scenarios [26].

We suggest that this unique SD architecture can be related to force distribution along the tooth. Occlusal contacts caused by compressive forces translate to sliding along wear facets, leading to tooth attrition. Various studies aimed to examine occlusal loading on single-rooted teeth exhibiting stress concentrated at the tooth cervix using FEA [26,27,28]. Benazzi et al. demonstrated tensile and compressive stress below the CEJ using an experimental model evaluating patterns of stress distribution under occlusal load simulated from different directions. Evaluating the various possible loading scenarios during mastication, and the combination of tensile and compressive stresses distributed along the root is evident, with compressive forces at 1–2 mm below the CEJ. Similarly, strain gauges indicated stress focusing below the CEJ, also leading to the formation of non-carious lesions [29,30]

In order to eliminate confounding factors, we used an anthropological collection which curated a relatively homogenous group of individuals with similar diet and lifestyle. However, this was also a limitation of the study as, together with the inclusion criteria (specific age and dental condition), restricted the sample size. However, considering the results of the study, out of the 30 teeth, 24 presented a thickening pattern of SD around the CEJ. The other six individuals presented thickening of the SD more apically, and no individual presented the maximal thickening in the crown area. This pattern was constantly repeated, indicating that the thickening was not directly related to the area of attrition; rather, it might have been related to force distribution along the tooth.

Unveiling the effect of attrition on SD formation has several clinical implications. Pulp obliteration may affect the ability to perform root canal irrigation and disinfection, and SD accumulation plays a significant role in this phenomenon [5,7]. In our sample, age-related accumulation was evident when compared to younger specimens, whereas attrition played a less significant role. Our data suggest that the age of the individual, rather than the attrition level, may affect the SD accumulation within the root canal more.

When considering the effect of our findings on age estimation methodologies, it should be noted that in cases of severe attrition, SD formation might constrict the pulp further and might increase age estimation.

We suggest that SD formation mechanism may be affected by occlusal loading, causing the activation of odontoblastic cells to form a more robust SD layer. Our findings indicate that dentin formation exhibits a dynamic buildup pattern, that might serve as a compensatory mechanism, possibly allowing for mechanical modulation of the tooth throughout life. This biological evidence may corroborate the FEA computed models.

## 5. Conclusions

The secondary dentin formation pattern was consistent regardless of the attrition level, showing thickening below the CEJ, mostly on the mesial and distal surfaces. The thickening area coinciding with maximal stress and strain distribution sites resulted from occlusal loadings. Unveiling the pattern of SD formation due to attrition might change our understanding of odontoblastic activity. Although classically regarded as affected by caries and aging, occlusal forces may also play a role in SD formation mechanism.

## Figures and Tables

**Table 1:** Age and attrition distribution of the studied sample. Molnar’s rank for attrition.

HTH	Age	Attrition	Tooth No.	Mid-Root SD/PD	Maximal SD/PD	Mesial	Buccal	Distal	Lingual
HTH 0920	60	1.5	45	0.07	0.16	0.59	0.42	0.60	0.44
HTH 2173	60	1.5	45	0.07	0.12	0.53	0.32	0.39	0.30
HTH 0265	50	2	35	0.08	0.14	0.73	0.49	0.74	0.47
HTH 2222	54	2	35	0.06	0.16	0.69	0.55	0.66	0.43
HTH 0966	50	2.5	35	0.08	0.15	0.79	0.59	0.79	0.54
HTH 1959	50	2.5	35	0.06	0.09	0.77	0.60	0.70	0.54
HTH 2006	59	2.5	35	0.05	0.11	0.69	0.51	0.74	0.61
HTH 0770	50	3	35	0.05	0.13	0.83	0.59	0.75	0.64
HTH 2211	50	3	45	0.08	0.14	0.47	0.31	0.49	0.38
HTH 2416	53	3	45	0.06	0.12	0.71	0.46	0.58	0.53
HTH 0537	55	3	45	0.07	0.16	0.75	0.59	0.81	0.64
HTH 1656	50	3	35	0.13	0.15	0.58	0.41	0.58	0.51
HTH 2560	52	3	34	0.06	0.13	0.63	0.51	0.65	0.40
HTH 2309	54	3	35	0.07	0.13	0.57	0.44	0.60	0.40
HTH 3393	54	3	45	0.09	0.15	0.74	0.46	0.69	0.50
HTH 3317	58	3	35	0.08	0.16	0.67	0.52	0.74	0.51
HTH 1796	60	3	45	0.10	0.16	0.58	0.42	0.60	0.43
HTH 0713	59	3.5	35	0.15	0.23	0.56	0.44	0.60	0.43
HTH 2089	59	3.5	45	0.06	0.11	0.77	0.58	0.75	0.53
HTH 1455	53	4	45	0.05	0.19	0.73	0.49	0.82	0.57
HTH 0401	54	4	34	0.07	0.12	0.63	0.52	0.63	0.41
HTH 2186	54	4	35	0.08	0.17	0.78	0.54	0.81	0.54
HTH 1904	55	4	35	0.10	0.16	0.74	0.46	0.72	0.48
HTH 1458	56	4	35	0.09	0.14	0.86	0.47	0.87	0.50
HTH 2312	53	4.5	35	0.07	0.17	0.82	0.57	0.80	0.57
HTH 0393	56	4.5	45	0.07	0.13	0.75	0.51	0.75	0.65
HTH 1561	52	5	35	0.11	0.21	0.67	0.38	0.55	0.34
HTH 0477	55	5	45	0.09	0.16	0.81	0.43	0.75	0.54
HTH 1236	55	6	35	0.09	0.22	0.74	0.36	0.72	0.46
HTH 3115	57	6	35	0.10	0.20	0.59	0.41	0.82	0.43

## Data Availability

Data was obtained from the Hamann-Todd Osteologic Collection (Laboratory of Physical Anthropology at the Cleveland Museum of Natural History, Cleveland, OH, USA. Hereinafter, HTH) and are available from the authors upon request with the permission of the Hamann-Todd Osteologic Collection manger.
